# Lingual artery thrombosis as a presentation of infective endocarditis in a pregnant patient: a case report

**DOI:** 10.1093/ehjcr/ytae550

**Published:** 2024-10-16

**Authors:** Martín Castillo, Francisca Hanuch, Geraldine Rauch, Patricio Avendaño, Oscar Cuevas

**Affiliations:** Department of Cardiology, Clínica Alemana de Santiago, Avenida Manquehue Norte #1499, 7650568 Vitacura, Chile; Department of Internal Medicine, Hospital Padre Hurtado, Esperanza 2150, 8880465 San Ramon, Chile; Department of Internal Medicine, Hospital Padre Hurtado, Esperanza 2150, 8880465 San Ramon, Chile; Department of Cardiology, Clínica Alemana de Santiago, Avenida Manquehue Norte #1499, 7650568 Vitacura, Chile; Department of Cardiology, Clínica Alemana de Santiago, Avenida Manquehue Norte #1499, 7650568 Vitacura, Chile

**Keywords:** Case report, Infective endocarditis, Pregnancy, Lingual artery thrombosis

## Abstract

**Background:**

Infective endocarditis during pregnancy is a rare condition that compromises the health of both the mother and the foetus, presenting high rates of morbidity and mortality. The clinical manifestations of this disease are varied, with embolic phenomena being a frequent presentation.

**Case summary:**

We report the case of a Hispanic 37-year-old patient, at 29 weeks of pregnancy, with no known cardiovascular history, who presented with 48 h of sudden mandibular and lingual pain. The study showed acute thrombosis of the right lingual artery and the rest of the right external carotid artery. In this context, searching for the origin of the embolism, acute mitral valve endocarditis was diagnosed, which was effectively treated with antibiotic therapy and biological mitral valve replacement, as well as early delivery.

**Discussion:**

We report the first case where lingual artery thrombosis was the key diagnostic feature of infective endocarditis.

**Learning points**

- Arterial embolism—even in unusual sites—may be the first overt sign of infective endocarditis, and its presence should raise clinical suspicion.
- Cardiologic conditions during pregnancy require a multidisciplinary team and expert consult guidance, as less data are available for evidence-based decision-making.

## Introduction

Infective endocarditis (IE) is an uncommon condition, with an incidence of 10/100 000 inhabitants per year and a 30-day mortality rate of up to 30%.^1^ In pregnancy, IE is less frequent, with calculated incidences of 0.006%.^2^ Despite not being identified as a risk factor for this pathology, pregnant patients are reported to have a mortality rate as high as 11%.^2,3^

Both the microbiological profile and the clinical presentation in pregnant women are the same as the one in the general population, although the presence of cardiac murmurs and anaemia—both common findings in endocarditis—may be present in healthy pregnant women. Additionally, around 25% of overall IE patients present some evidence of embolism upon admission,^1^ which has been shown to be higher in pregnant women, reaching up to 50% of cases in some reports.^2–4^

## Case presentation

We present the case of a 37-year-old Hispanic female, in her 29th week of pregnancy, with no significant past medical history, who consulted for 48 h of right mandibular and lingual pain. Besides her pain, she did not experience fever, chest pain, or shortness of breath. On interrogation, she reported a febrile episode 2 months prior to the event associated with arthralgias, which spontaneously resolved. There was no history of night sweats, weight loss, or other symptoms. No dental or invasive procedures were performed in the past 12 months.

**Figure 1:**
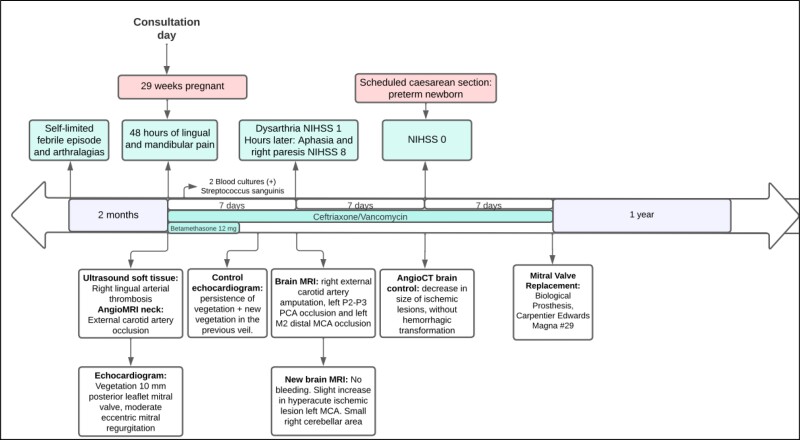
Timeline. NIHSS, National Institutes of Health Stroke Scale; PCA, posterior cerebral artery; MCA, middle cerebral artery; MRI, magnetic resonance imaging; P2-P3, P2-P3 segment of posterior cerebral artery; M2, M2 segment of middle cerebral artery.

On examination, the patient was alert and in no distress. Her temperature was 37.2°C; her pulse was regular, with a rate of 112 b.p.m.; and a soft pansystolic murmur was audible over the left fifth intercostal space, midclavicular line, that radiated towards the axilla. There was no sign of heart failure or infection (*Figure 1*).

The haemoglobin was 9.8 g/dL with a leucocyte count of 14 800/cu.mm: 86% were neutrophils and the erythrocyte sedimentation rate was 62. C-reactive protein value was of 9.8 mg/dL.

The biochemical parameters were glucose 84 mg/dL, creatinine 1.1 mg/dL, bilirubin 1.2 mg/dL, serum glutamic oxaloacetic transaminase 24 mg/dL, serum glutamate pyruvate transaminase 30 mg/dL, and cholesterol 178 mg/dL.

**Figure 2:**
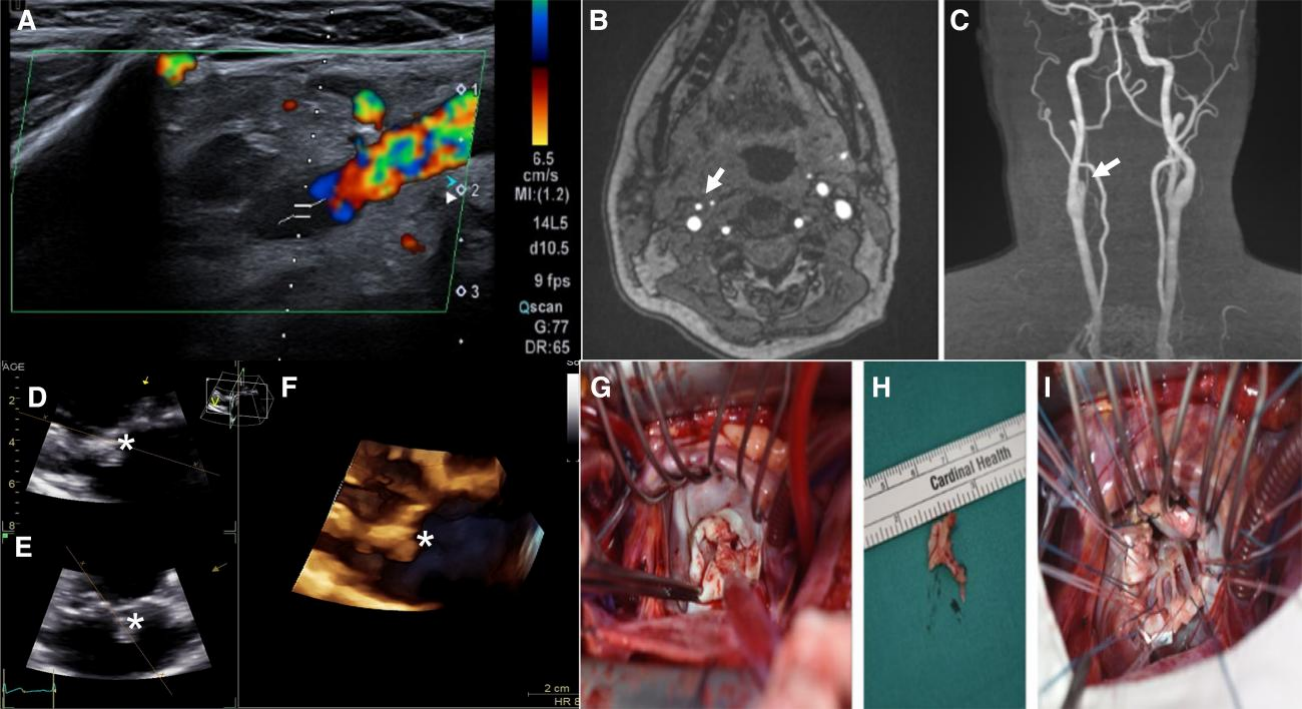
Central figure. (*A*) Doppler ultrasound of the lingual artery; absence of flow in the evaluated segment of the right lingual artery. (*B* and *C*) Magnetic resonance imaging angiography of the neck and brain vessels. Transverse and coronal sections demonstrate asymmetry in the representation of the external carotid artery (white arrow). (*D–H*) Echocardiogram showing a vegetations on the atrial side of the mitral valve (*). (*F*) 3D modelling of the 2D images. (*G–H*) Cardiac surgery. (*G*) Exploration of the mitral valve with diffuse involvement of the valve, featuring a prominent vegetation at the A2-A3 junction. (*H*) Surgical specimen of the coaptation area of both leaflets with large vegetations. (*I*) Mitral valve replacement with a bioprosthetic valve.

An ultrasound study of the mouth showed thrombosis of the right lingual artery (*Figure 2A*), and a neck magnetic resonance imaging (MRI) angiography revealed an occlusion of the right external carotid artery (*Figure 2B* and *C*).

Infective endocarditis was suspected, and the echocardiogram confirmed a 10 mm vegetation on the posterior leaflet of the mitral valve, with moderate mitral regurgitation (*Figure 2D–F*) (Supplementary material online, *Video S1*).

Blood cultures were collected, and antibiotic treatment was initiated empirically, with ceftriaxone and vancomycin. The patient was admitted in the coronary care unit.

The blood cultures were positive for penicillin susceptible *Streptococcus sanguinis* in all samples, and imaging studies ruled out embolic events in other organs, including the fetoplacental unit. After susceptibility rates were confirmed, treatment was continued with ceftriaxone.

Odontogenic infective focus was ruled out by maxillofacial evaluation, and a foetal lung maturation protocol was performed with betamethasone.

After 5 days of treatment, the follow-up echocardiogram showed persistence of the vegetation on the posterior leaflet, with no changes in its size, and a new vegetation on the anterior leaflet, measuring 6 × 4 mm, as well as persistence of moderate mitral regurgitation.

A week after admission, the patient presented an episode of mild dysarthria, which was classified as National Institutes of Health Stroke Scale (NIHSS) 1. The brain MRI revealed occlusion of the left posterior cerebral artery and occlusion of the left middle cerebral artery in its distal M2 portion. A few hours after the episode, the patient developed aphasia and right facial paresis, with a NIHSS score rising to 8 points. A second MRI ruled out bleeding and showed a slight increase in the size of the hyperacute ischaemic lesion in the superficial territory of the left middle cerebral artery and no changes in the other ischaemic lesions.

As thrombolysis is contraindicated during pregnancy, she was managed with neuroprotective measures.

After the neurological events, and with lung maturation already achieved, a C-section was scheduled, during which a preterm newborn was delivered without complications. After the procedure, the patient had a favourable obstetric and neurological outcome, recovering all her previous functions.

Two weeks after the stroke, the patient underwent mitral valve replacement with a bioprosthetic valve (*Figure 2I*). During the examination of the mitral valve, vegetations in both leaflets were described, as well as infiltration of the entire coaptation surface and part of the primary chordae tendineae (*Figure 2G* and *H*). Histopathological examination of the valve suggested incipient myxomatous disease. The patient had a satisfactory postoperative course without complications.

At the 2-year follow-up, the patient and her son continue to have a favourable evolution. The patient has a normal neurological examination and normal prosthetic valve function in all her echocardiograms.

## Discussion

Infective endocarditis is an uncommon condition in the general population and even less frequent during pregnancy as aforementioned, often presenting with mild symptoms, making it a diagnostic challenge.

Acute onset of jaw and tongue pain led to the diagnosis of lingual artery thrombosis, and though not being a classic feature, IE was suspected and diagnosed. The lingual artery corresponds to an uncommon site of thrombosis and normally in this setting thrombophilia must be ruled out. In this case though, no search for coagulation disorders was performed, since pregnancy itself is a prothrombotic state, to which acute endocarditis was associated, providing sufficient factors for thromboembolic events to occur.

Cardiac diseases complicate around 1% of pregnancies.^5^ The recently updated European Society of Cardiology guidelines^6^ suggest that the management of endocarditis in pregnancy should be the same as in the general population, with antibiotic treatment adjusted to isolated microbiology, and preferring always those that are approved for administration during pregnancy. The indication for urgent surgery remains cardiogenic shock or refractory heart failure due to severe acute valve regurgitation. Nonetheless, when surgery is required for uncontrolled infection, i.e. the appearance of new septic embolisms despite antibiotic treatment, as was the case of our patient, the foetal and maternal risk should be evaluated on a case-by-case basis, and whenever possible, extraction of the viable foetus should be attempted before cardiac surgery, due to the high foetal mortality that exists when the foetus is exposed to cardiopulmonary bypass.^7^

This is the first case reported in the literature of IE presenting with mandibular–lingual pain, adding a new symptom to the myriads of clinical presentations of this disease. The few reported cases of mycotic aneurysms of the external carotid arteries normally present with neurological symptoms or a pulsatile mass.^8^

Pregnancy is often perceived as a foreign ground for clinical cardiologists, with less evidence available for decision-making. Also stroke complicating IE limited our timeframe for surgical intervention, thus imposing another challenge to this odd scenario.

Multidisciplinary evaluation, experts consult, and patient opinion must all be balanced in the treatment of this patient, and we believe that our case report could help teams in acting on likewise scenarios in the future.

## Conclusion

Mycotic aneurysms of the external carotid artery are a rare form of presentation of IE. We report the only case known by the authors of thromboembolism of the lingual artery as a manifestation of IE.

## Supplementary Material

ytae550_Supplementary_Data

## Data Availability

The data that support the findings of this case report are available on request from the corresponding author. The data are not publicly available because it contains information that could compromise the privacy of the patient.

## Lead author biography

Dr Martín Castillo, MD, hails from Santiago, Chile. He obtained his MD degree from Universidad Del Desarrollo and pursued further studies at the same institution, specializing in internal medicine and subsequently in cardiology. Currently undertaking a fellowship in echocardiography, he serves as cardiology staff at Clínica Alemana de Santiago. Beyond medicine, Dr Castillo is an avid sports enthusiast and a passionate aficionado of classical music, having nurtured his musical talents since childhood.

## Supplementary material

Video of the echocardiogram; Parasternal long axis view of the mitral valve with a vegetation on the atrial side. Supplementary material is available at *European Heart Journal – Case Reports* online.
